# SWOT analysis of the dental hygiene profession in Pakistan—past, present, and future

**DOI:** 10.1038/s41405-024-00255-y

**Published:** 2024-09-05

**Authors:** Iqra Damani, Shazia Taimoor, Fahad Umer, Rashna Hoshang Sukhia, Ali Sadiq

**Affiliations:** Section of Dentistry, Department of Surgery, Aga Khan University, Stadium Road, Karachi, PO Box 3500 Pakistan

**Keywords:** Dentistry, Preventive dentistry

## Abstract

**Background:**

Pakistan faces a significant burden of oral diseases, which can be effectively reduced through preventive measures. Dentistry in Pakistan predominantly focuses on corrective dental procedures, increasing the treatment costs and widens disparities in oral healthcare access. To address this gap and meet the country’s oral health needs, Aga Khan University initiated a Dental Hygiene program aimed to expand and diversify the oral health workforce and improving access to quality care in various healthcare settings. Due to limited awareness of this profession in the country, the program encounters significant challenges.

**Aim:**

This article aims to present a SWOT analysis of the Dental Hygiene profession in Pakistan and propose evidence-based strategic changes to address these challenges and improve future outcomes.

**Methods:**

A SWOT analysis was conducted to identify the internal strengths, weaknesses, external opportunities, and threats related to the Dental Hygiene profession, gathering both quantitative and qualitative data through a survey of relevant stakeholders (Consultants, Dental hygiene graduates, dental auxiliaries, fresh dental graduates, and prospective students) using Research Electronic Data Capture (REDCap).

**Results:**

A total of 267 respondents participated in the survey, providing insights into the current state of the Dental Hygiene profession.

**Conclusion:**

The analysis reveals that the Dental Hygiene profession in Pakistan requires robust advocacy, increased collaboration with dentists, opportunities for higher education, and the establishment of proper legislative frameworks to prevent professional transgression beyond the scope of practice.

## Introduction

Noncommunicable Diseases (NCDs) are a major healthcare burden worldwide [1]. In oral health, Dental caries and Periodontal disease are two primary NCDs globally [2]. According to systematic reviews conducted in 2021 and 2022, the prevalence of Dental caries is more than 60% and Periodontal Disease is 56% across all provinces of Pakistan [3, 4]. Untreated carious lesions can be painful and may lead to functional limitation [3]. Conversely, untreated periodontal disease along with having intraoral consequences is correlated with other systemic diseases such as stroke, cardiovascular disease, and preterm birth in pregnant women [5]. The incidence of oral disease in Pakistan compounds the significant burden of NCDs [6]. Additionally, there is a high prevalence of oral cancer and oral submucous fibrosis [7]. Thus, the oral disease burden in Pakistan is notably high.

Dentistry in Pakistan predominantly focuses on corrective procedures such as drilling and filling. This approach increases treatment costs and widens oral healthcare disparities. According to Khan 2019, less than 3% of treatments in government dental institutions were preventive services such as examination, scaling, and prophylaxis, while over 90% were tooth extractions [8, 9]. The practicing dentist-to-population ratios (DPR) vary widely among countries. As per WHO, the DPR in most Western countries is 1:2000. As per 2018 estimates, the DPR for Pakistan was around 1:10,000. This shows the DPR to remain far below the accepted value [8]. Furthermore, the country has a dire insufficient supply of quality dental professionals. According to 2018 estimates, only 21,000 licensed dentists are registered with the Pakistan Medical Commission (PMC), which is inadequate for a population of over 235 million people. The unmet oral needs of the population are intensified further as oral diseases become more progressive and cumulative, increasing the complexity of issues over time [8].

The Dental Hygiene program in Pakistan was initiated by the Aga Khan University (AKU) to address this gap. The aim of the vocation is to expand and diversify the oral health workforce, enabling access to quality care in the public and private sectors. Regular checkups by these well-trained professionals can prevent painful and severe dental problems and help in early detection of oral health issues, including pre-cancerous lesions [5].

To date, fifty-one students have graduated with a Diploma in Dental Hygiene (DDH), which was later upgraded into the Associate of Science in Dental Hygiene (ASDH). The graduates provide services in primary, secondary and tertiary healthcare settings, private dental clinics, academia, and research. Till 2022, this program had reached 300,000 people through community outreach activities. (https://www.aku.edu/mcpk/undergraduate/Documents/Dental%20Hygiene%20Program%20Report%202022.pdf).

But not being a widely known profession to the population, this vocation faces several challenges such as acceptance from dentists and community, absence of legislation and regulatory body, and avenues of completing higher education. To systematically identify the strengths, weaknesses, opportunities, and threats a SWOT analysis was conducted to address and mitigate the challenges faced by this profession.

## Materials and methods

The study was approved by the Aga Khan University Ethical Review Committee, ERC number 2023-9467-27282.

This study explicitly focused on relevant stakeholders involved with the Dental Hygiene Profession. The five stakeholders identified were the AKU dental hygiene graduates, dental consultants, dental auxiliaries (dental assistants and technicians), fresh dental graduates and prospective students. The survey forms were created on the software Research Electronic Data Capture (REDCap). The sample size was calculated as considering a population size (Dentist, Dental Auxiliaries, Alumni, Dental Graduates, and Prospective students) of 5000, with a hypothesized percentage frequency of outcome factor in the population of 50%, a confidence limit of 5%, and a design effect of, a total sample size of 357 individuals will be required to achieve a confidence level of 95%.

### Data collection

All the survey administration and data collection were done online. The participants were approached through the existing program office database, social media groups, and word of mouth. The e-consent was taken from all the participants at the beginning of the survey. Intense efforts were made to ensure the representation of participants from diverse geographic regions within Pakistan to capture a comprehensive perspective. Participant’s selection criteria are provided in Supplementary Table 1.

## Results

The results are presented in five sections: first, the results from AKU dental hygiene graduates; second, the responses of dental consultants; third, the responses of dental auxiliaries; fourth, the responses of final-year dental students/house officers; and fifth, the prospective students.

**Fig. 1:**
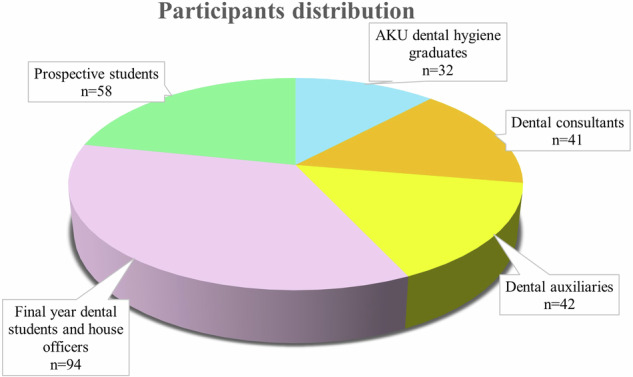
Participants distribution. illustrate the participants distribution across all groups. *n* = 32 AKU dental hygiene graduates, *n* = 42 dental auxiliaries, *n* = 41 dental consultants, *n* = 94 final year dental students and house officers, and *n* = 58 prospective students.

A total of *n* = 267 respondents participated in the survey. The study included five different surveys which were distributed among relevant stakeholders. This included *n* = 32 AKU dental hygiene graduates, *n* = 42 dental auxiliaries, *n* = 41 dental consultants, *n* = 94 final year dental students and house officers, and *n* = 58 prospective students (refer Fig. 1).

### AKU dental hygiene graduates

In this survey, 32 AKU dental graduates participated. Of these, 60% (*n* = 19) reported working as Dental Hygienists, 18% (*n* = 6) were working in other fields (such as business analysts, research associates, and program officers), and 13% (*n* = 4) were pursuing higher education. Only 9% (*n* = 3) were neither working nor studying. Additionally, 94.3% (*n* = 29) expressed a strong desire to pursue higher education.

On a scale of 1–10, the mean rating for the overall quality of the dental hygiene academic program was 6.73, while the mean satisfaction level with the program’s clinical training and experience was 7.27. Furthermore, 51.3% (*n* = 19) agreed that they were happy with their career choice in dental hygiene. 21.6% (*n* = 8) were not happy with dental hygiene as their career choice, where 27% (*n* = 10) remained neutral about it.

A notable 72% of dental hygiene graduates who responded highlighted the need for licensing and regulation of the profession in Pakistan. The survey also revealed that 10.8% (*n* = 4) strongly disagreed and 29.7% (*n* = 11) were neutral about their ability to apply their dental hygiene education in their current workplace. Lastly, 48.6% strongly believed that additional training and education are needed to enhance their skills as dental hygienists. 83.8% (*n* = 31) strongly advocated for four years degree instead of two years (refer Table 1).

**Table 1:** Open-ended responses of AKU dental hygiene graduate survey.

What did you find most challenging after graduating from AKU Dental hygiene programme?
*“People not recognizing what dental hygienists are and do. It’s a new profession so this kind of challenge is what we face. not finding opportunities to work in clinic and then finding it hard to enroll in a course for further education.”*
*“Making the population accept that we exist too and play crucial role in healthcare system.”*
*“Explaining my role as a dental hygienist to the potential employers who were not familiar with this job at all.”*

Open-Ended Responses of AKU Dental hygiene graduates on challenges they faced after completing dental hygiene degree in Pakistan.

### Dental consultants

This survey included *n* = 41 consultant dentists from various specialties (Prosthodontist, Periodontist, Orthodontist, Endodontist, and Maxillofacial Surgeon) representing both private and institutional practices; 92.8% (*n* = 37) agreed that dental hygienists contribute positively to their practice and 87.8% (*n* = 35) acknowledged their role in revenue generation, with 68.3% (*n* = 27) willing to hire dental hygienists, while 24.4% (*n* = 10) were not sure.

However, 12.1% (*n* = 5) consultants also raised a concern on professional transgression beyond the scope of practice as a threat. Other highlighted points in the survey were reported as economic feasibility 2.4% (*n* = 1), collaboration between dental hygienists and dentists 2.4% (*n* = 1) and need for dental hygiene regulatory body in Pakistan 4.8% (*n* = 2). (refer Table 2) (Supplementary Table 2).

**Table 2:** Open-ended responses of dental consultant survey.

“In your opinion, how can we make this collaboration (dentist and hygienist) better?”
*“Introducing dental hygiene programs and producing high quality, clinically and market ready dental hygiene graduates.”*
*“Encourage people on taking hygienist as a profession and awareness amongst dentists about the role and utilization of hygienist. their role and advantages.”*
*“A national set-up that allows collaboration of the two where none thinks the other can take their monetary share. this is achieved by defining the goals for dental hygienists.”*
“*There is a need to develop guidelines and legislation to help regulate the vocation*.”

Open-Ended Responses from Dental Consultants on increasing collaboration between dentist and dental hygienist.

### Dental auxiliaries (dental assistants/nurses and dental hygienists)

65% (*n* = 26) dental auxiliaries were not aware of the dental hygiene profession. However, after the survey a considerable proportion of the respondents 73.2% (*n* = 30) showed a desire to further their careers by pursuing dental hygiene. Furthermore, 76% (*n* = 29) of dental assistants showed interest in enrolling in a four-year bachelor’s program instead of a two-year associate degree (Supplementary Table 3).

### Final-year dental students and house officers

Among the 94 fresh dental graduates and students, 71.7% (*n* = 29) expressed interest in pursuing an Associate of Science in Dental Hygiene (ASDH). 94.3% (*n* = 88) of dental students and house officers showed interest in higher education, supporting the introduction of advanced educational programs, and 85.7% (*n* = 80) of final year dental students advocated for a bachelor’s degree in dental hygiene (Supplementary Table 4).

### Prospective students

There was *n* = 58 respondents for the prospective student’s survey. Pre-medical high school students accounted for 75.4% (*n* = 43) of the respondents, and 58.6% (*n* = 34) of this subgroup were not aware of the dental hygiene program/profession. In terms of dental hygiene degree preferences, 19.0% (*n* = 11) stated they had no preference, 36.2% (*n* = 21) preferred an Associate of Science in Dental Hygiene, while 44.8% (*n* = 26) preferred a Bachelor of Science in Dental Hygiene. 15.5% (*n* = 9) of responded that they would be willing to consider a new career despite limited job opportunities, 44.8% (*n* = 26) said that they would choose a career with secure job prospects, and 39.7% (*n* = 23) responded that they were unsure.

The findings of the SWOT analysis are summarized in Table 3, providing a comprehensive overview of the results.

**Table 3:** SWOT analysis.

Strengths	Weakness
**Based on Other Stake Holders**	
▪ Consultants acknowledge the role of dental hygienist in patient care and revenue generation	▪ Lack of awareness about the profession among dental auxiliaries and prospective students
▪ Focus on disease prevention	▪ Economic feasibility of integrating dental hygienist into private practices.
▪ Consultants willing to hire dental hygienists	▪ Limited career growth opportunity in current healthcare system
▪ Expansion and diversification of workforce, reducing disease burden	▪ Absence of regulatory body
▪ Options to diversify in academics, public health, and research	
**Based on AKU Dental Hygiene Graduate Study**	
▪ The majority of AKU dental hygiene graduates working as a Dental hygienist	▪ A few gradates not content with their career choice
▪ High desire among respondents to pursue higher education	▪ Recognition of profession in the country
▪ High satisfaction with clinical training and experience	▪ Some respondent felt they could not apply their education in their current workplace

Opportunities	Threats
▪ Strong inclination to study a four-year degree program	▪ Switching of profession
▪ Potential for career progression in different fields	▪ Lack of licensing and regulation poses a risk to professional credibility and standards
▪ Career progression for dental auxiliary	▪ Lack of oral healthcare infrastructure in rural areas
▪ Potential to improve oral health prevention and reduce disease burden	▪ Job market saturation in urban areas
▪ Collaboration with dentist and other dental auxiliaries	▪ Domain transgression beyond scope of practice
▪ Advocacy for the creation of a regulatory body	▪ Current economic climate of country

Strengths, weakness, opportunities, and threats of dental hygiene profession in Pakistan.

## Discussion

According to WHO, the overall NCD burden in Pakistan is high (https://www.who.int/publications/m/item/oral-health-pak-2022-country-profile) [9]. The current healthcare system faces several challenges, including lack of access and resources [10]. This is further compounded by the fact that there is heavy emphasis on curative procedures rather than prevention. A recent white paper by the European Federation of Periodontology (EFP) recommended diversifying the oral health workforce by including dental hygienists and therapists to tackle the disease burden [2]. Strengthening the dental hygiene profession, which heavily focuses on prevention, could reduce the strain on tertiary care facilities and decrease the incidence of oral diseases [11]. Oral diseases like dental caries, gingivitis, periodontitis, and oral cancers are preventable through increased public education and awareness initiatives, oral health therapies and regular oral screenings [11]. In countries with successful dental hygiene programs there has been a notable decline in oral disease [12].

An analysis of the dental hygiene profession in Pakistan incorporated insights from five key stakeholder groups to evaluate its strengths, weaknesses, opportunities, and threats. AKU dental hygiene graduates provided firsthand insights into professional challenges, while dental consultants offered crucial perspectives since dental hygienists must work under their supervision. Dental assistants, as core members of dental practices, represent potential candidates for career progression into dental hygiene. Additionally, dental students, house officers, and prospective students contributed valuable insights into external opportunities and the profession’s future potential. This comprehensive understanding of the current state and future possibilities for dental hygiene in Pakistan was achieved using a SWOT approach, chosen for its broad narrative overview [13]. A systematic review was not considered due to the limited number of relevant articles and diverse, state-specific nature of global dental hygiene literature, with no specific studies on Pakistan. Therefore, the most meaningful insights come from stakeholders discussing the profession’s past, present, and future in Pakistan.

The AKU dental hygiene graduates survey identified that the majority of the graduates (60%) are working as Dental Hygienists, which shows a strong initial uptake of the profession. Additionally, the presence of graduates working in other fields (18%) or pursuing further education (13%) suggests a more diverse career pathway and opportunities within this field. This pattern is also seen in North American and European countries where this profession is more established. A study conducted in Canada reported that 45% respondents worked outside the traditional clinic practice settings, specifically in education, public health, and research [14].

The dental hygiene profession in Pakistan still is in infancy and lacks awareness and acceptability among the population and healthcare providers. This may contribute to the divided opinions on career satisfaction, with 51.3% content with their choice, while 27% remain neutral and 21.6% are dissatisfied. Additionally, the lack of awareness of other career pathways also leads to dissatisfaction. In the dental hygiene survey, respondents expressed concerns about the associate degree (two-years), highlighting the need for a bachelor’s degree (four-year) as it is more known and acceptable in Pakistan.

Both the dental consultant and dental hygiene surveys highlighted the strong need for licensing and regulatory bodies. Like National Health Services (NHS), the licensing and regulatory body in Pakistan will help in bringing this profession in the National healthcare framework. It will also help in standardizing and monitoring the scope of practice of dental hygiene. Such body would provide guidelines and Standard Operating Procedures (SoP), preventing professional domain transgression beyond the scope of practice and giving the profession the required legitimacy. Furthermore, this will create societal trust and create positive impact on job opportunities for dental hygienists.

In our survey, dental consultants acknowledged the positive contribution of dental hygienists in dental care. The inclusion of dental hygienist allows dentists to focus on more complex procedures, enhancing the overall effectiveness and efficiency of dental care delivery. This model ensures comprehensive care that addresses both preventive and curative needs, improving overall patient outcomes [15, 16].

One of the biggest threats to the dental hygiene profession is the lack of awareness among prospective students about the field. This lack of awareness is hindering the profession’s growth and sustainability. Therefore, strong advocacy is essential for this profession to put its foot in society and become a viable career choice. This can be done via educational campaigns in high school, promotion through mainstream media, and social media. Strong advocacy is important to sustain this profession. (The study’s findings are synthesized in Table 3, providing a comprehensive overview of the key results discussed).

The progression of dental hygiene profession can also help mitigate one of the Pakistan’s longstanding healthcare challenges: malpractice and quackery. Many untrained dental professionals (quacks), who are practicing without any formal education in dentistry [17, 18]. Treatment from these un-trained quacks often lead to serious transmissible infectious diseases such as human immunodeficiency virus/acquired immunodeficiency syndrome, Hepatitis B, and C [18]. People often resort to these quacks because they are inexpensive and often only accessible option in certain areas, especially rural regions. In many of these rural areas, there is a complete lack of formal oral healthcare infrastructure. Implementing effective use of qualified dental hygienists in primary, secondary, tertiary care practices would be highly beneficial. Placing them in these centers could enhance the practice of by provision of basic dental care services, while referring patients with advanced conditions to qualified dentists. This preventive approach aims to have a long-term positive effect on the overall health status of society at large.

### Strengths of the study

According to our research, no previous study has examined the dental profession in Pakistan in this manner. This study involved multiple stakeholders to identify the strengths, weaknesses, opportunities, and threats facing the profession. It serves as a foundational analysis to reflect on and address the challenges within Pakistan’s dental sector. Additionally, this study provides evidence-based insights that can inform policy changes and help predict future outcomes.

### Limitations of the study

The findings related to AKU dental hygiene graduates cannot be generalized to all practicing dental hygienists. Although the survey forms were distributed to participants nationwide, the forms did not collect information about the respondents’ provinces, which would have allowed for a more accurate provincial representation. There was response bias, as only the most motivated individuals participated in the survey. Therefore, it may not accurately reflect the views of all hygienists, consultants, and dental auxiliaries. Furthermore, there was sampling bias, as not all population segments had equal access to the internet or the survey platform. Only thirty two out of fifty-one AKU dental hygiene graduates responded.

## Conclusion

In our study, we identified four key factors crucial to advancing the dental hygiene profession in Pakistan: career pathways, legalization and regulatory body, scope of practice, and advocacy. Establishing clear and diverse career pathways is essential for professional growth and attracting talent to the field. Implementing robust legal frameworks and establishing a dedicated regulatory body are necessary to standardize practices, ensure quality, and protect both practitioners and patients. Defining and expanding the scope of practice for dental hygienists can enhance their role within the healthcare system, enabling them to perform a broader range of preventive and therapeutic procedures. Promoting advocacy efforts to raise awareness about the importance of dental hygiene and the contributions of dental hygienists can help garner support from policymakers, stakeholders, and the public. Addressing these factors can lead to significant improvements in the dental hygiene profession in Pakistan, ultimately contributing to better oral health outcomes and overall public health.

## Supplementary information

Supplementary Information

## Data Availability

The data analyzed during the current study are available from the corresponding author upon reasonable request.
