# Supplementary material for: SWOT analysis of the dental hygiene profession in Pakistan—past, present, and future
Source: BDJ Open. 2024 Sep 5;10:71. doi: 10.1038/s41405-024-00255-y (PMC11377419; doi:10.1038/s41405-024-00255-y)
Supplement: Supplementary file 1 — Supplementary Information [file 41405_2024_255_MOESM1_ESM.pdf]

Supplementary Table 1: Participant Selection Criteria

**Inclusion criteria**	
AKU dental hygiene graduates	Participants who have graduated from the Aga Khan University dental hygiene Program.
Dental Consultants	Dental professionals with at least five years of experience run a dental practice or work with the organization
Dental Assistants and Auxiliaries	Individuals working as dental assistants or technologists
Dental students and house officers	• Individuals who are in their last year of bachelor's in dental surgery (BDS).<br>• Individuals who have recently completed BDS in Pakistan.<br>• House officers
Prospective students	Highschool students with majors in sciences or premedical (compulsory biology as a subject)
**Exclusion criteria**	
All groups	Participants unwilling to participate in the study.

Results

Supplementary Table 2: Dental Consultants Results

Variable		Frequency n=41	Percentage
Currently practicing as a dentist?	Yes	39	95.1
	No	2	4.9
Area of Specialty	General Dentist	9	22
	Periodontist	14	34.1
	Prosthodontist	5	12.2
	Endodontist	6	14.6
	Maxillofacial Surgeon	4	9.8
	Orthodontist	3	7.3
	Dental Office	8	19.5

Private clinic or work within an organization	Works with an organization	18	43.9
	Both	15	36.6
Dental Hygienist contribute positively to patient care	Strongly agree	19	46.3
	Agree	19	46.3
	Neutral	3	7.3
	Disagree	0	0
	Strongly Disagree	0	0
Dental Hygienist contribute to revenue generation	Strongly agree	12	29.3
	Agree	24	58.5
	Neutral	4	9.8
	Disagree	1	2.4
	Strongly Disagree	0	0
Dentist and dental hygienist collaboration	Strong collaboration with clear roles & responsibilities	4	9.8
	Some collaboration, but roles & responsibilities not very well defined	14	34.1
	Very limited	13	31.7
	No collaboration	10	24.4
Willingness to hire a dental hygienist	Yes	28	68.3
	No	3	7.3
	Not Sure	10	24.4

Supplementary Table 3: Dental Auxiliaries Results

Variable		Frequency n=41	Percentage
Gender	Male	25	61.0
	Female	14	34.1
	No response	2	4.9
Educational Background	Matriculation	2	4.9
	F. Sc Pre-med	16	39.0
	FA<br>Arts (FA)	1	2.4
	Others	18	43.9
	No Response	4	9.8
Formal education related to dental hygiene or auxiliary	Yes	31	75.6
	No	7	18.4
	No Response	3	7.3
Aware of ASDH at AKU	Yes	26	63.4
	No	13	31.7
	No Response	2	4.9
Currently employed	Yes	24	58.5
	No	15	36.6
	No Response	2	4.9
DH/DA certification	Yes	21	51.2
	No	18	43.9
	No Response	2	4.9
Current professional status satisfaction	Happy with current status	13	31.7
	Seeking career progression	26	63.4
	No Response	2	4.9
Would like to pursue Dental	Yes	30	73.2
	No	9	22.0
Hygiene	No Response	2	4.9
Associates or Bachelor's?	Associates	9	22
	Bachelor's	28	68.3
	No Response	4	9.8

Supplementary Table 4: Fresh dental graduates & house officers results

Variable		Frequency n=94	Percentage
Dental graduate or student	Graduate	64	68.1
	Student	30	31.9
Employment status	Yes, I'm working	51	54.3
	No, I'm completing my BDS	23	24.5
	No, I'm seeking job	10	10.6
	No, preparing for further studies	6	6.4
	No Response	4	4.3
Dentist or house officer	Dentist	20	21.3
	House officer	38	40.4
	None	36	38.3
